# On the Influence of Chloroform in Promoting Cutaneous Absorption

**Published:** 1870-03

**Authors:** 


### On the Influence of Chloroform in Promoting Cutaneous Absorption.

From a number of experiments detailed in the *Practitioner* for December, 1869, Dr. Augustus Waller, of Geneva, deduces the following :

1. Chloroformic solutions applied to the skin of man and animals are quickly absorbed, and produce local and general results according to the substances employed.

2. Alcoholic and aqueous solutions are either not at all or very slowly absorbed.

3. Chloroform easily traverses the dead skin by diffusion.

4. Alcohol does not traverse the skin, but produces an endosmotic current with water.

5. Skin exposed to chloroform in a state of liquid or vapor absorbs a considerable quantity of it.

6. On traversing the septal skin of the endosmometer, chloroform carries with it a certain amount of any alkaloid disolved in it.

7. These observations sufficiently explain the rapidity of cutaneous absorption during life, without our having recourse to any problematic influence of sebaceous matter on the surface of the skin.

In the course of his experiments, Dr. W. discovered that it was necessary for cutaneous absorption that the heart's action be not in any way diminished, for if it be, no absorption takes place, even after a prolonged immersion. This is owing not so much to the absence of local absorption in the skin, as to the deficiency in the vascular action; for if we remove the skin of a part that has been immersed, we can detect in its inner surface the presence of the dissolved substance, which, however, from the deficient means of transport in the vessels around, has remained localized and inert in the system.

